# In mice transgenic for IGF1 under keratin-14 promoter, lifespan is decreased and the rates of aging and thymus involution are accelerated

**DOI:** 10.18632/aging.101903

**Published:** 2019-04-13

**Authors:** Vladimir N. Anisimov, Irina F. Labunets, Irina G. Popovich, Margarita L. Tyndyk, Maria N. Yurova, Alexey G. Golubev

**Affiliations:** 1 Department of Carcinogenesis and Oncogerontology, N.N. Petrov National Medical Research Center of Oncology, Saint Petersburg 197758, Russia; 2 Laboratory of Experimental Models, State Institute of Genetic and Regenerative Medicine, National Academy of Medical Sciences of Ukraine, Kiev 04114, Ukraine

**Keywords:** aging, IGF1, thymus, thymulin, papilloma, cancer, survival pattern, parametric analysis, Gompertz model, Strehler-Mildvan correlation, age-related diseases

## Abstract

IGF1 signaling is supposedly a key lifespan determinant in metazoans. However, controversial lifespan data were obtained with different means used to modify IGF1 or its receptor (IGF1R) expression in mice. The emerging puzzle lacks pieces of evidence needed to construct a coherent picture. We add to the available evidence by using the Gompertz model (GM), with account for the artifactual component of the Strehler-Mildvan correlation between its parameters, to compare the survival patterns of female FVB/N and FVB/N-derived K14/mIGF1 mice. In K14/mIGF1 vs. FVB/N mice, the rate of aging (*γ*) is markedly increased without concomitant changes in the initial mortality (*μ*_0_). In published cases where IGF1 signaling was altered by modifying liver or muscle IGF1 or whole body IGF1R expression, lifespan changes are attributable to *μ*_0_. The accelerated aging and associated tumor yield in K14/mIGF1 mice are consistent with the finding that the age-associated decreases in thymus weight and serum thymulin are accelerated in K14/mIGF1 mice. Our results underscore the importance of accounting for the mathematical artifacts of data fitting to GM in attempts to resolve discrepancies in survival data and to differentiate the contributions of the initial mortality and the rate of aging to changes in lifespan.

## Introduction

Within species or strain comparisons suggest that, in mammals that feature reduced signaling through the key components of the somatotropic neuroendocrine axis, i.e. growth hormone (GH) and insulin-like growth factor-1 (IGF1), lifespan is increased, tumor incidence is decreased, and aging may be slowed down [1,2]. Mounting data suggest that these effects are mediated through developmental early-life body tuning [3,4]. Later in life, IGF1 deficiency may be adverse by predisposing, in particular, to sarcopenia [5], myocardial insufficiency [6], and neurodegeneration [7]. Indeed, transgenic male FVB/N mice, in which IGF1 overexpression under α-myosin heavy chain promoter is confined in adults to the heart and skeletal musculature, not only feature a range of beneficial changes in the heart but also live significantly longer while having serum IGF1 level twofold higher than in controls [8]. In male but not female mice in which IGF1 production in the liver is downregulated at adult ages, lifespan is decreased and tumor incidence is increased [9,10].

The conundrum of beneficial vs. adverse effects of increased IGF1 signaling on lifespan is further complicated by reports about the gender- and- strain-specific effects of IGF1 receptor underexpression in mice [11–13].

The available evidence is insufficient to combine properly the known pieces of the puzzle that thus has emerged. Here we provide additional pieces by reporting on changes in lifespan and tumor incidence that accompany IGF1 expression driven by keratin-14 promoter in FVB/N-derived transgenic K14/mIGF1 female mice. The local beneficial effects of IGF1 overexpression in the skin of these mice are manifested as enhanced wound healing [14]. Increased IGF1 expression in the skin of K14/mIGF1 mice was reported, but no changes in serum IGF1 levels, as well in heart and liver weights, were found in K14/mIGF1 vs. FVB/N mice [14]. This suggests that the effects of IGF1 in K14/mIGF1 mice are not systemic but rather are confined to the sites of IGF1 overexpression. These sites are not limited to the skin in K14/mIGF1 mice, since keratin-14, whose gene promoter is used to drive IGF1 expression in this case, is expressed also in the reproductive system and the upper gastrointestinal tract (https://www.proteinatlas.org/ENSG00000186847-KRT14/tissue) and in the thymus [15].

It is increasingly recognized that a major source of inconsistencies in aging research is the lack of attention to differences between the two main contributors to the lifespans of study cohorts: the initial viability, and the rate of its age-dependent decline (the rate of aging). These two contributors are captured by the Gompertz model (GM), which is commonly used in aging research for analyzing survival and mortality patterns [16–20]:

$$\mu (t)=\frac{dN(t)}{dt}\times \frac{1}{N(t)}=\mu_{0}\cdot e^{\gamma t},$$

where: *μ*(*t*) is the force of mortality; *N*(*t*) is the number of survivors at age *t*; *μ*_0_ is the initial mortality, which may be interpreted as capturing, in an inverse manner, the initial viability, and *γ* is the rate of aging.

In virtually none of the original publications about the effects of changes in IGF1 signaling on lifespan, attempts were made to differentiate the contributions of changes in the two GM parameters to the observed effects. This gap is filled in the present paper by comparing changes in the estimates of *γ* and *μ*_0_ found upon comparing of K14/mIGF1 and wild type (WT) FVB/N mice and derived from published survival curves related to mice having experimentally induced alterations in IGF1 signaling. This was done with account for the recently recognized artifactual correlation between the two GM parameters [20–22].

## RESULTS

### Original findings and discussion thereof

**Figure 1:**
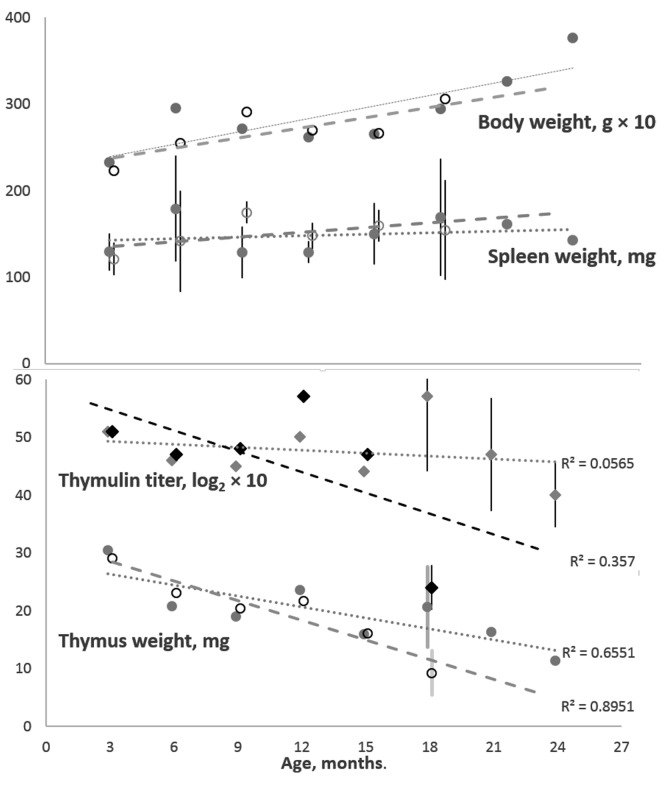
**Body and spleen weights (the upper panel) and thymus weight and serum thymulin titer (the lower panel) in the cohorts of female K14/mIGF1 and WT FVB/N mice.** Body weight and thymulin titer data are multiplied by 10 to better accommodate all data to the respective panels. To avoid encumbering, shown are only the 95% confidence intervals that suggest significant differences between K14/mIGF1 and WT FVB/N mice (the lower panel) and the lack of significant differences in the spleen weights of the two strains (the upper panel). K14/mIGF1 mice are presented with dashed lines and open markers, except for thymulin data markers (filled black diamonds). FVB/N mice are presented with dotted lines and filled gray markers. No comparison between K14/mIGF1 and FVB/N data are possible after ages above 18 months because the former mice do not live thus long.

Figure 1 shows that, in the cohort of female K14/mIGF1 mice compared with WT FVB/N mice, an accelerated age-dependent thymus involution is paralleled by an accelerated age-dependent decrease in serum thymulin titer. The divergences between the trends seen in the two cohorts lead to initially small differences, which increase to statistical significance at later ages. At the same time, body weights and their changes with age are virtually identical in both cohorts, so as spleen weights, the latter being constant through the adult lifespan.

Since keratin-14 is known to be expressed in the thymus [15] (but not in the spleen and lymph nodes (https://www.proteinatlas.org/ENSG00000186847-KRT14/tissue) it is reasonable to hypothesize that IGF1 gene expression driven by keratin-14 promoter takes place in the thymus, not only in the skin of K14/mIGF1 mice. No direct data about IFG1 expression in the thymus, unlike the skin [14], of K14/mIGF1 mice are available so far. However, the expression of both IGF1 and IGF1R in unmodified murine and human thymus epithelium has been demonstrated [23,24]. Thus, if the local IGF1 expression in the thymus is altered being driven by keratin-14 promoter, the alteration is likely to produce effects via IGF1R. Our finding that the effects are manifested as accelerated thymus involution is quite counterintuitive since exogenous IGF1 administration has been shown [25,26] to increase thymus weight by expanding thymus epithelium.

**Figure 2:**
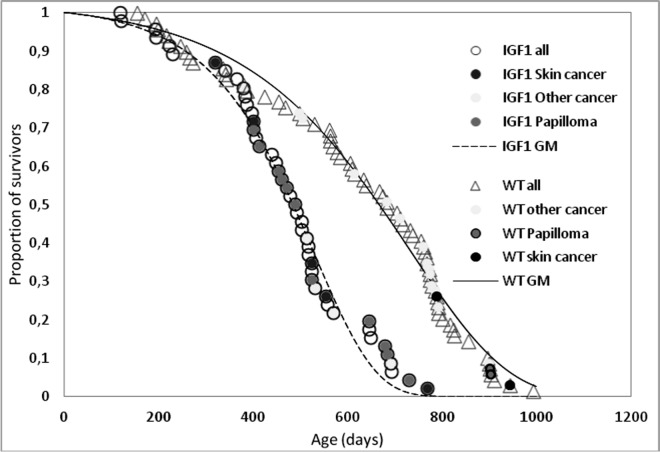
**Survival patterns of K14/IGF1 mice (IGF1, dashed line) and WT FVB mice (solid line).** Data points associated with postmortem tumor detection are shade-coded. Smooth lines show survival data approximations with the Gomperz model (GM).

Whatever the cause of the accelerated thymus involution in K14/mIGF1 mice, their survival pattern is consistent with accelerated aging as suggested by the survival curves of K14/mIGF1 and WT FVB/N mice, which are compared in Figure 2. The numerical estimates of their lifespan and survival parameters are presented in Table 1.

**Table 1:** Survival parameters in female WT FVB/N and K14/IGF1 mice.

		WT FVB/N **(*N*_0_ = 69)**	K14/mIGF1 **(*N*_0_ = 46)**	Difference
Maximum lifespan, days		993	770	–22.5%
Median lifespan, days		683	484	–29%
Mean lifespan, days (mean±SD)	All	622±227	469±156	–25%, p<0.01
	The last 10% survivors	921±36	714±35	–22%, p<0.01
Initial mortality *μ*_0_, days^–1^ (95% CI)		0.00014 (0.00011; 0.00016)	0.00013 (0.00009; 0.00017)	0%
Aging rate *γ*, days^–1^ (95% CI)		0.0049 (0.0046; 0.0052)	0.0077 (0.0069; 0.0084)*	+57%, p<0.05

The involvement of thymus involution in aging and in the development of age-associated immunologic and neuroendocrine disorders in mice is long recognized (reviewed in [27–29]). However, the available evidence mostly shows how aging influences the thymus. There is very limited if any experimental data showing how interferences with the rate of thymus involution are translated into changes in the rate of aging.

The causes of accelerated thymus involution in K14/mIGF1 mice must relate somehow to IGF1 expression driven by keratin-14 promoter. Serum IGF1 has been shown to be unaltered in K14/mIGF1 compared with WT FVB/N mice [14]. However, even if serum IGF1 were increased because of IGF1 overexpression driven by keratin-14 promoter, which is known to take place in the skin of K14/mIGF1 mice [14], still the available evidence [25,26] is consistent with that, in adults, this would enhance thymic functions and increase lifespan. Therefore, the accelerated aging of K14/mIGF1 mice is likely associated with not the systemic effects of altered IGF1 signaling but rather with the accelerated thymus involution. Indirect evidence (see above) suggests the hypothesis that the acceleration may relate to the local IGF1 expression driven by keratin-14 promoter in thymus epithelial cells.

Another feature of K14/mIGF1 compared with WT FVB/N mice is the increased incidence of skin papillomata (Table 2). Increased susceptibility to papilloma was also observed in the transgenic mice that express IGF1 under keratin-1 or keratin-5 gene promoter [30,31]. The distributions of papillomata and other spontaneous tumors over the lifespans of K14/mIGF1 and WT FVB/N mice is shown with shade-coded points in Figure 2.

**Table 2:** Spontaneous tumors found in the cohorts of female K14/mIGF1 and WT FVB/N mice over their whole lifespans.

**Tumor**	**The numbers of mice found postmortem to have a defined tumor**	
	**WT FVB/N *N*__0__ = 69)**	**K14/mIGF1 *(N*__0__ = 46)**
**Skin**	**4 (5.8%)**	**17 (34.7%)^a^**
Papilloma	2	12^a^
Squamous cell carcinoma	2	5
**Other sites**	**17 (24.5%)**	**7 (17.3%)**
Harderian gland cystadenosarcoma cystadenocarcinoma	1	0
Lung adenocarcinoma	9	2
Lung adenoma^b^	0	1
Lung carcinosarcoma	0	1
Lymphoma	5	0
Mammary adenocarcinoma	0	1
Mesenterial angioma^b^	0	1
Ovarian serous cysts^b^	1	0
Uterine angiosarcoma	0	1
Uterine granulosa cell tumor^b^	1	0
**All tumors except for papilloma**	**19 (27.5%)**	**12 (26%)**

^a^ p<0.01 (exact Fisher test)

^b^ benign tumors

An increase in the lifelong prevalence of skin carcinomas in K14/mIGF1 vs. FVB/N mice is conspicuous but statistically insignificant, so as other differences in lifelong oncological indices between the two strains. A noteworthy observation is the predominance of lung cancer among tumors found in WT FVB/N mice, which is consistent with findings noted by other authors [32]. A lowered proportion of lung carcinomas among all tumors in K14/mIGF1 mice is insignificant statistically.

**Figure 3:**
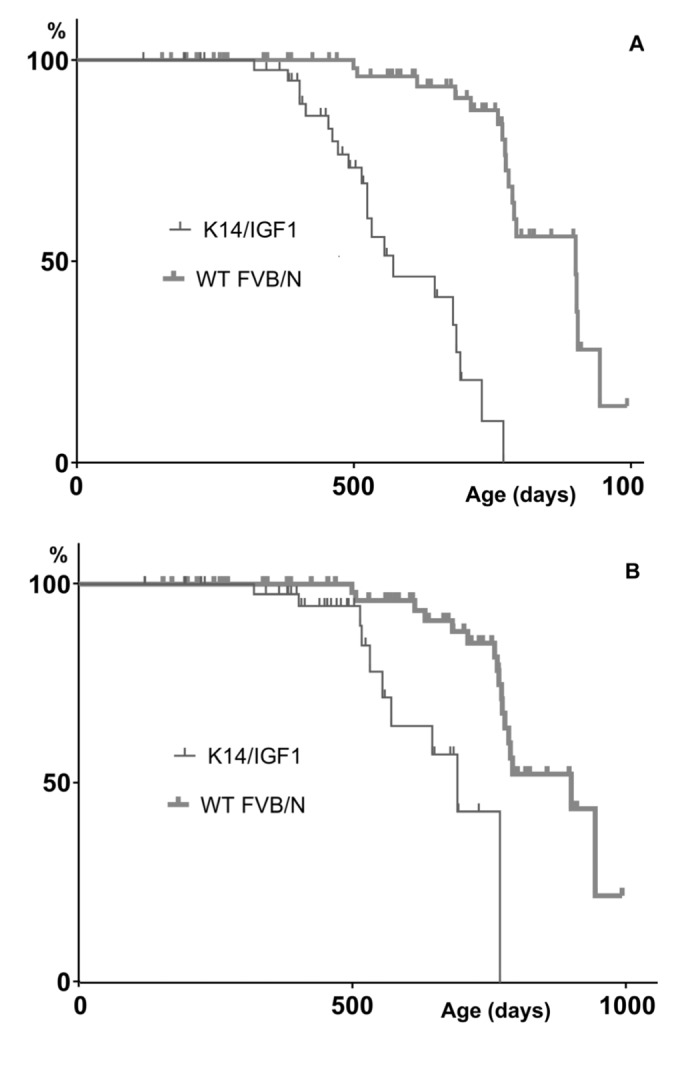
**Kaplan-Meyer plots constructed upon censoring for the death cases that are not associated with any postmortem tumor detection**. (**A**) or not associated with a postmortem tumor other than papilloma (**B**).

Further insight into relationships between spontaneous carcinogenesis and aging may be obtained by comparing survival patterns upon their censoring for cases not associated with postmortem tumor detection. The results are presented in Figure 3.

In the presence case, the Kaplan-Meyer plots show the probabilities of not dying without having a detectable tumor by a certain age. These probabilities decrease when tumors are initiated earlier and/or grow faster. This is what occurs in K14/mIGF1 mice as follows from that differences between censored plots related to K14/mIGF1 and WT FVB/N mice are significant according to the log-rank test (p<0.001) irrespective of whether papillomata are taken into account.

Taken together, our findings suggest that, in mice, the acceleration of thymus involution and of the associated decrease in serum thymulin will shorten lifespan because of accelerated aging rather than increased initial mortality rate (Table 1 and Figure 2). The accelerated aging is manifested, in particular, in accelerated tumor initiation and/or growth (Figure 3). This conclusion *per se* does not depend on any assumptions concerning the cause of accelerated thymus involution. In the present case, the cause must relate somehow to IGF1 expression driven by keratin-14 promoter. Below it will be shown that the present case of modified IGF1 signaling differs drastically, with regard to murine survival patterns, from published cases of altered IGF1 signaling where either other promoters were used to modify IGF1 expression or IGF1R expression was altered.

### Comparative analysis of original and published survival data

Differentiating changes in the initial viability, which in the Gompertz model (GM) is captured, in an inverse manner, by the initial mortality *μ*_0_, and in the rate of its decline (the rate of aging, *γ*) is essential for understanding the effects of interventions into lifespan [17,19,20]. It will be shown below that, in this regard, the K14/mIGF1 model is strikingly different from other models used to study the effects of interventions in IGF1 signaling on lifespan in mice.

**Figure 4:**
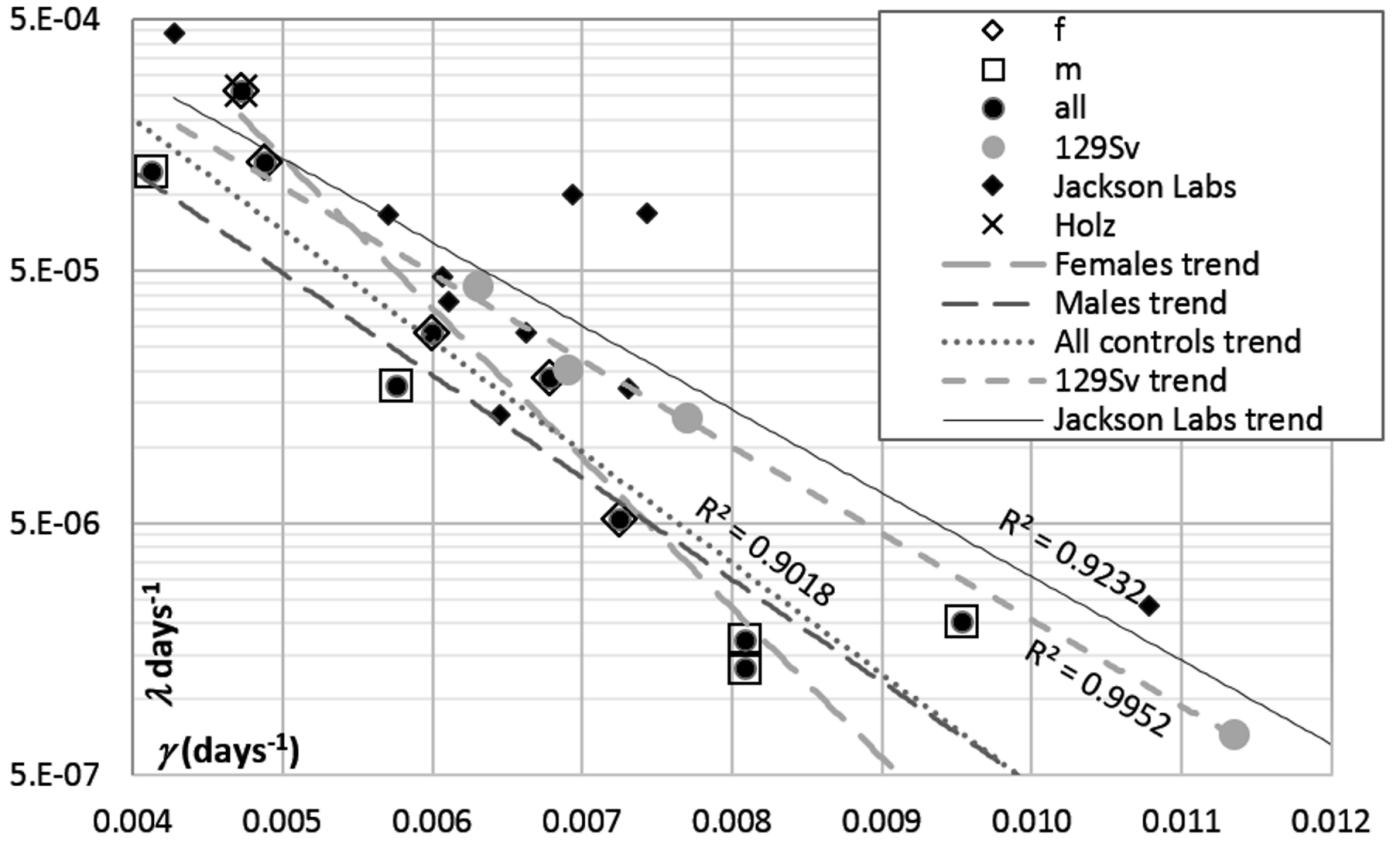
**Correlations between GM parameters (*μ*_0_ and *γ*) derived from the survival curves of:** (**i**) control mice used in IGF1 signaling experiments performed in different labs (see references to Figure 5), (**ii**) female mice bread at Jackson Laboratories, and (**iii**) several samples of female 129/Sv mice bread at authors’ laboratory [20]. The cross (Holz) in the upper left corner highlights the point related to female 129/Sv mice studied by Holzenberger et al. [12] and confirms the general consistency of the female 129/Sv trend, which is shown with gray round markers.

**Figure 5:**
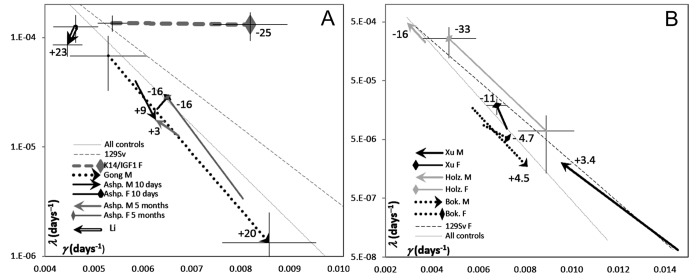
**The plots of *μ*_0_ (log scale) vs. *γ* (linear scale) estimates derived from survival curves presented in publications where the effects of modification of IGF1 signaling in adults were reported.** The heads of vectors are directed towards increased IGF1 signaling. The numbers at vector heads show reported percent changes in the median lifespans. Thin lines show the reference regressions of *μ* on *γ*, related to controls described in these publications and to 129/Sv mice (the same as in Figure 4). Crosshairs show 95% CI for the estimates of GM parameters. To avoid cluttering, crosshairs are added only to selected data points that illustrate the whole range of the CIs. (**A**) Different promoters were used to modify IGF1 expression. Hammerheads: decreased lifespans. Arrows: increased lifespans. References: Gong [10]; Li [8]; Ashp [9]. (**B**) IGF1R^++^ and IGF1R^+-^ mice were compared. Hammerheads: females. Arrows: males. References: Holz [12]; Bok [11]; Xu [34].

The artifactual component of correlation between GM parameters [20–22] makes it a tricky matter to distinguish the contributions of changes in the initial mortality and in the rate of aging to changes in lifespan. Figure 4 shows the estimates of *μ*_0_ (logarithmic ordinate) vs. *γ* (linear abscissa) extracted from published survival curves related to the parent (control) strains of mice used to alter IGF1 signaling by gene engineering (references are provided in the legend to Figure 5). A linear correlation between ln*μ*_0_ and *γ* is apparent. However, the respective regression of ln*μ*_0_ on *γ* is almost parallel to the two other regressions shown in Figure 4, one of which is derived from data on several murine strains bred in a single laboratory (http://www.jax.org/research-and-faculty/research-labs/the-harrison-lab/gerontology/available-data), and the other, from data on several samples of female 129/Sv mice all drawn from a single general population [20]. The latter regression is 100% artifactual. Therefore, the other two must comprise significant artifactual components.

To distinguish between real and apparent changes in GM parameters, we employed the approach suggested and thoroughly discussed in [20], where it was used to differentiate the effects of calorie restriction and of drugs believed to mimic it. In essence, when a change in *μ*_0_ is associated with a reciprocal change in *γ*, the latter is valid only if it is different from what the artifactual regression of ln*μ*_0_ on *γ* would suggest. Ideally, for each particular population of a specific murine strain and gender, this regression must be derived from a series of control samples (as it is in the case of 129/Sv mice in Figure 4) or the randomization of a sufficiently large control sample into several subsamples. However, because all regression lines in Figure 4 are almost parallel, their general trend may be used as a reference.

Applying this approach to data about transgenic mice in which IGF1 signaling is modified in different ways (Figure 5) suggests that the difference between K14/mIGF1 and WT FVB/N mice is unique in that the respective vector (thick dashed gray line at the top of Figure 5A) is parallel to the *γ* axis. In this regard, the K14/mIGF1 vs. WT FVB/N case stands out of all other cases irrespective of a possible interpretation of the patterns presented in Figure 5. A plausible interpretation is that the decrease in the lifespan of K14/mIGF1 mice is completely attributable to accelerated aging. The magnitude of the decrease is reflected more by the deviation of the vector from the slope that reflects the artifactual relationships between the estimates of GM parameters, rather than by the length of the vector. Among all cases analyzed in this way earlier [20], only calorie restriction was associated with that the orientations of the respective vectors were almost parallel to the *γ* axis, albeit the direction of the vectors was reciprocal to that related to K14/mIGF1 vs. WT FVB/N mice. Calorie restriction is generally believed to slow down aging. This observation strengthens the conclusion that accelerated aging is largely responsible for the significant decrease in the lifespan of K14/mIGF1 mice.

Another case of a significant deviation of the corresponding vector from the artifactual slope relates to mice in which IGF1 overexpression is confined to the heart and muscles and, with that, is associated with increased circulating IGF1 [8], that is with enhanced systemic IGF1 effects. A marked increase in the median lifespan in this case (the thick hollow vector in the top left corner of Figure 5A) is brought about by decreases in both, the initial mortality (*μ*_0_) and the rate of aging (*γ*). As far as the respective vector clearly deviates from the reference (artifactual) regression, changes in both of the parameters must be real and unidirectional and, thus, must be associated with a marked change in lifespan, although the vector is much shorter than that related to K14/IGF1 vs. WT FVB/N mice. Because the effects of increased IGF1 signaling brought about by elevated blood IGF1 must be beneficial for the thymus [25,26], decelerated aging in this case may be partially attributed to decelerated thymus involution, whereas decreased initial mortality, to the systemic effects of increased IGF1 signaling. The latter proposal is prompted by the observation that the systemic changes in IGF1 signaling that are brought about by interferences with IGF1R expression influence *μ*_0_ rather than γ (see Figure 5B and its discussion below).

The third case of a marked deviation of changes in GM parameters from the artifactual relationships between them is presented by data of Ashpole et al. [9]. In female mice, IGF1 downregulation at the age of 10 days increases lifespan, which is equivalent to a decrease upon upregulation, as reflected by the direction of the respective vector head in Figure 5A. This black hammerhead vector is almost perpendicular to the artifactual slope, this being consistent with increases in both, *μ*_0_ and *γ*. When the same intervention starts at 5 months of age, a similar decrease in the median lifespan (considered as if occurring upon increased IGF1 signaling) is accountable for by increased *μ*_0_. The slope of the relatively long gray hammerhead vector being close to the artifactual slope. In male mice, lifespans seem to increase upon increasing IGF1 signaling; however, the effects are small and, in terms of changes in GM parameters, are contradictory, the corresponding reciprocally directed vectors being almost parallel to the artifactual slope.

In [10], a significant decrease in lifespan upon downregulation of IGF1expression in the liver of male mice was reported. This is equivalent to an increase in lifespan upon IGF1 up-regulation (relative to, perhaps, an inappropriately low level). The respective (dotted) vector in Figure 5A suggests that the increase is caused by a decrease in *μ*_0_, whereas the apparent increase in *γ* (which *per se* could not increase lifespan) is largely artifactual as far as the vector is parallel to the reference regression, which is completely artifactual. The magnitude of the artifact, as well as of 95% confidence limits of the estimates of GM parameters may relate to the small numbers of animals (15 and 16) in the groups compared. There was no effect on lifespan in females in this study, and the female survival plot presented in [10] is too messy to extract numerical data from it.

In Figure 5B, IGF1R^++^ and IGF1R^+–^ mice are compared. The latter are transgenic, and the former are wild type, that is, the former are regarded as controls. However, because in the present context the consequences of enhanced IGF1 signaling is the matter of interest, all vectors shown in Figure 5B are directed towards points related to control instead of transgenic mice. Even without a detailed analysis, it is clear that the orientations of the vectors shown in Figure 5B are overtly different from the vector related to K14/mIGF1 mice in Figure 5A, no matter how the patterns seen in Figure 5 may be interpreted. Some suggestions concerning Figure 5B interpretation are given below

The gray vectors in Figure 5B relate to data reported in [12]. The authors demonstrated significant increases in the lifespan of 129/Sv mice in which systemic IGF1 signaling was attenuated by gene engineering resulting in IGF1R haploinsufficiency (IGF1R^+–^). This is equivalent to decreased lifespans in IGF1R^++^ vs. IGF1R^+–^ mice. The effect is more expressed in females. Because lifespan cannot be decreased by the deceleration of aging *per se*, the primary effect must be a real increase in *μ*_0_ associated with an apparent decrease in *γ*, which is largely artifactual since it is not greater than the reference regressions suggest.

When the same gene-engineering approach was applied to C56BL mice, the conclusion was that upon attenuated IGF1 signaling, lifespan is increased in females only and is decreased in males [11]. Dotted vectors in Figure 5B show these effects in a reciprocal manner. However, since the effects are small, within 5%, they must be, most likely, irreproducible according to the “probability of detection” criterion introduced in [33]. In Figure 5B, the respective vectors are short and almost parallel to the reference regressions. It was confirmed subsequently [34] that decreased IGF1 signaling increases lifespan only in the females of C56BL mice. This effect is shown in Figure 5B with the black hammerhead vector. The magnitude of the effect is consistent with the marked deviation of this relatively short vector from the reference regressions. The decrease in lifespan is attributable to increased *μ*_0_, whereas the apparent increase in *γ* is distinctly smaller than the reference regressions suggest. As to the lifespan of C56BL males according to data extracted from survival curves presented in [34], it seems not to depend on changes in IGF1 signaling within ranges possible upon IGF1R haploinsufficiency. Indeed, the respective vector (black arrowhead), although it is long, suggests that a decrease in *μ*_0_ is associated with a greater than it follows from the reference regressions decrease in *γ*; that is, an apparent increase in the estimate of the initial mortality is compensated by an apparent decrease in the estimate of the rate of aging. The associated alteration of lifespan is so small that little, if anything, more than artifactual is presented by this case.

## DISCUSSION

In the final account, the comparison of survival patterns reported in the literature and in the present work suggests that, in female mice, increases in the systemic effects of IGF1 signaling are usually associated with decreases in lifespan. In male mice, on the contrary, increases in lifespan are more prevalent, although they are less expressed and consistent. It is important that in both, males and females, changes in the initial mortality *μ*_0_ are the main contributors to the observed effects.

Not surprisingly, the effects of IGF1 overexpression strongly depend on the promoters that drive it (compare [8] and the present work, Figure 5A). Surprising is our finding that when IGF1 expression, which is known to take place in the thymus [23,24], is additionally driven by the promoter of the keratin-14 gene, age-associated thymus involution and serum thymulin decrease become accelerated. The acceleration is apparent even against the background of wild type FVB/N mice, which have been shown to feature premature thymus involution and reduced lifespan compared with those of C56BL/6 mice [35].

Notably, in ln*μ*_0_ vs. *γ* plots, the vector related to K14/mIGF1 vs. wild type FVB/N mice is parallel to the *γ* axis, which is strikingly different from all other vectors in Figs. 5. This difference is remarkable *per se* irrespective of its possible interpretations. Calorie restriction is the only other interference in lifespan, among several other interferences whose results have been treated in the same way [20] as here, to be consistently manifested as long horizontal vectors in ln*μ*_0_ vs. *γ* plots. Calorie restriction is generally believed to slow down aging rather that to decrease the initial mortality. This strengthens the conclusion that increased aging rate is what makes the lifespan of K14/mIGF1 mice to decrease.

The association of this effect of IGF1 expression driven by keratin-14 promoter with accelerated thymus involution is quite unexpected in view that the exogenous IGF1 administration (and, by inference, increased systemic IGF1 signaling) is known to expand the epithelial component of the thymus [25]. IGF1R is expressed in thymic epithelial cells [24,36]. In mice, age-related decline in thymic function parallels the decline of blood plasma IGF1 [37]. Administration of exogenous IGF1 is associated with increased thymic mass and enhanced thymic function in mice [25]. Therefore, even if serum IGF1 were increased in our K14/mIGF1 mice (although no increase in this strain was reported earlier by Semenova et al. [14]), this would not accelerate thymus involution.

There are no direct data concerning IGF1 expression in the thymus of K14/mIGF1 mice; however, the available evidence cited above suggests that intracellular IGF1 may be expressed inappropriately in the epithelial cells of their thymuses, including the cells that produce thymulin.

Interestingly, it has been shown, albeit in pigs, that IGF1R is more highly expressed in the medulla of juvenile thymus compared with aged thymus and that IGF1R-positive cells express keratin 14 [38]; hence, IGF1 gene under keratin 14 promoter must be expressed in the thymus and act via its receptor.

Whatever the cause of the accelerated decreases in thymulin level and thymus weight in aging K14/mIGF1 mice, their rate of aging rather than initial mortality is increased. The increased aging rate is shown here to be associated with accelerated tumor initiation and/or promotion and/or growth. The last two options seem more likely because of the involvement of the thymus in the immunological surveillance. Thus, the unique role of thymus as a determinant of spontaneous carcinogenesis and aging in mice is underscored.

Ironically, virtually nothing is known still about the molecular targets of one of the main secretory products of the thymus, i.e. thymulin, and about the mechanisms of its production [39], although almost half a century has passed since thymulin had been discovered. However, numerous interrelationships between thymus secretory products, including thymulin, and the components of the immunoneuroendocrine system are known [27]. Therefore, an accelerated attenuation of thymus functions because of whatever cause, which in the present case relates to IGF1 expression driven by keratin-14 promoter, is likely to decrease lifespan by accelerating the age-dependent decline of the resistance of mice to numerous causes of death, including tumors, that is, by accelerating the rate of aging.

Notably, a recent publication [40] has strengthened the evidence that thymus involution is largely responsible for increasing cancer incidence in aging humans.

## MATERIALS AND METHODS

### Animals

Wild-type female FVB/N (WT FVB/N) and FVB/N-derived transgenic K14/mIGF1 mice [14] were donated by European Molecular Biology Laboratory Mouse Biology Unit (Monterotondo-Scalo, Rome, Italy). The mice were then housed and bred in parallel at the animal facilities of N.N. Petrov National Medical Research Center of Oncology (Saint Petersburg, Russia) and Institute of Genetic and Regenerative Medicine (Kiev, Ukraine). Cohort were established by combining animals born within one week after the date that preceded by three months the onset of the follow-up of a cohort. Five to seven mice were kept in polypropylene cages under a standard light/dark schedule (12:12) at 22±2°C with food and water *ad libitum*. When the numbers of mice in cohorts decreased because of deaths, the survived mice were randomly transferred to a smaller number of cages to keep 5 to 7 mice per cage throughout the whole study period, except for the last survivors. All mice were under veterinary control. All procedures were performed in conformance with European Convention for the Protection of Vertebral Animals Used for Experimental and Other Scientific Purposes, CETS No. 123, and were approved by the Ethic Committees of N.N. Petrov National Medical Research Center of Oncology and Institute of Genetic and Regenerative Medicine.

### Thymus and thymulin studies

To determine changes in thymus and spleen weights and serum thymulin titers and in whole body weight, the cohorts of female WT FBN/N mice (*N*_0_=77) and K14/mIGF1 mice (*N*_0_=57) were established at Institute of Genetic and Regenerative Medicine. Ten mice of each cohort were sacrificed by decapitation at the age of 3 months and in every 3 months thereafter. Thymuses and spleens were dissected and weighed. Serum samples obtained by centrifugation of clotted blood were stored at –20°C. Thawed samples were filtered through Centriflo CF-25A ultrafilter (Amicon, USA) to remove the high molecular inhibitor of thymulin. Thymulin titers were determined as described in [41]. The determination method is based on the ability of thymulin to restore the sensitivity of spontaneous rosette formation by splenocytes from thymectomized mice to inhibition with azathioprine (Sigma, USA). The results were recorded as log_2_(thymulin titer).

### Survival and carcinogenesis studies

To determine the survival patterns of mice, WT FVB/N (*N*_0_=69) and K14/mIGF1 (*N*_0_=46) cohorts were followed-up at N.N. Petrov National Medical Research Center of Oncology until the natural deaths of their last members. The related procedures have been described earlier [42]. All dead animals were autopsied. Any tumors as well as tissues and organs with a suspected tumor mass were excised and fixed in 10% neutral formalin. After routine histological processing, the tissues were embedded in paraffin. Five to seven 7-mm thick histological sections were stained with hematoxylin and eosin and examined microscopically. Tumors were classified according to International Agency for Research on Cancer.

### Survival analysis

Experimental and published survival data were approximated, as described in [20], with the parametric Gompertz model (GM):

$$\begin{array}{l} n(t)=\underset{\Delta t\to 0}{\lim}\frac{N(t)-N(t+\Delta t)}{\Delta t}=\frac{dN(t)}{dt},\\ \mu =n(t)\times \frac{1}{N(t)}=\mu_{0}\cdot e^{\gamma t}, \end{array}$$

where: *N*(*t*) is the number of survivors at age *t*; Δ*t* is the increment of *t*, *n*(*t*) is the decrement of *N*(*t*) during Δ*t*, *μ*(*t*) is the force of mortality, i.e., the decrement of *N*(*t*) during Δ*t* relative to the number of survivors at age *t*; *μ*_0_ is the initial mortality, which may be interpreted as capturing, in the inverse manner, the initial viability, and *γ* is the rate of aging.

Under these premises,

$$N(t)=N_{0}-{\text{e}}^{-\frac{\mu_{0}}{\gamma }({\text{e}}^{\gamma \cdot t}-1)}$$.

The basic GM model, which does not account for the age-independent mortality and for study sample heterogeneity, is appropriate when experimental samples are small [16,20]. The maximum likelihood method implemented in the TableCurve2D software tool ver. 5.1 (Systat Software Inc**.**, San Jose, CA, fully functional trial version available at http://www.sigmaplot.co.uk/products/tablecurve2d/tablecurve2d.php) was used to fit survival data to the above equations.

To account for the artifacts that are inevitably introduced to *μ*_0_ and *γ* estimates by any technique of survival data fitting to GM [22], the estimates were checked against the artifactual correlation between them as suggested in [20]. GM parameters were compared based on the overlaps of their 95% confidence intervals (CI).

### Statistics

Subsets of time series data related to small age intervals were treated using Mann-Whitney test. Contingency data were compared using the exact Fisher test and/or Chi^2^. Tabulated data are presented as means ± standard errors of the mean (with 95% CI in round brackets).

To get insight into the contribution of tumors to mortality, the Kaplan-Meier approach was applied: survival data were censored for cases not associated with the presence of a tumor upon postmortem examination. This was carried out using GraphPad Prism ver.7 software (GraphPad Software, San Diego, CA).
